# Knockdown of microglial iron import gene, DMT1, worsens cognitive function and alters microglial transcriptional landscape in a sex-specific manner in the APP/PS1 model of Alzheimer’s disease

**DOI:** 10.21203/rs.3.rs-4559940/v1

**Published:** 2024-06-27

**Authors:** Katrina Volk Robertson, Alec S. Rodriguez, Jean-Philippe Cartailler, Shristi Shrestha, Kyle R. Schroeder, Arianna M. Valenti, Fiona E. Harrison, Alyssa H. Hasty

**Affiliations:** Vanderbilt University; Vanderbilt University; Vanderbilt University; Vanderbilt University; Vanderbilt University; Vanderbilt University; Vanderbilt University Medical Center; Vanderbilt University

**Keywords:** microglia, iron, inflammation, DMT1, Slc11a2, Alzheimer’s disease, APP/PS1, neuroinflammation, sex differences, behavior

## Abstract

**Background:**

Microglial cell iron load and inflammatory activation are significant hallmarks of late-stage Alzheimer’s disease (AD). *In vitro*, microglia preferentially upregulate the iron importer, divalent metal transporter 1 (DMT1, gene name *Slc11a2*) in response to inflammatory stimuli, and excess iron can augment cellular inflammation, suggesting a feed-forward loop between iron import mechanisms and inflammatory signaling. However, it is not understood whether microglial iron import mechanisms directly contribute to inflammatory signaling and chronic disease *in vivo*. These studies determined the effects of microglial-specific knockdown of *Slc11a2* on AD-related cognitive decline and microglial transcriptional phenotype.

**Methods:**

*In vitro* experiments and RT-qPCR were used to assess a role for DMT1 in amyloid-β-associated inflammation. To determine the effects of microglial *Slc11a2* knockdown on AD-related phenotypes *in vivo*, triple-transgenic *Cx3cr1*^*Cre − ERT2*^;*Slc11a2*^flfl^;*APP/PS1*^+^^or −^ mice were generated and administered corn oil or tamoxifen to induce knockdown at 5–6 months of age. Both sexes underwent behavioral analyses to assess cognition and memory (12–15 months of age). Hippocampal CD11b + microglia were magnetically isolated from female mice (15–17 months) and bulk RNA-sequencing analysis was conducted.

**Results:**

DMT1 inhibition *in vitro* robustly decreased Aβ-induced inflammatory gene expression and cellular iron levels in conditions of excess iron. *In vivo*, *Slc11a2*^KD^ *APP/PS1* female, but not male, mice displayed a significant worsening of memory function in Morris water maze and a fear conditioning assay, along with significant hyperactivity compared to control WT and *APP/PS1* mice. Hippocampal microglia from *Slc11a2*^KD^ *APP/PS1* females displayed significant increases in *Enpp2*, *Ttr*, and the iron-export gene, *Slc40a1*, compared to control *APP/PS1* cells. *Slc11a2*^KD^ cells from *APP/PS1* females also exhibited decreased expression of markers associated with disease-associated microglia (DAMs), such as *Apoe*, *Ctsb, Csf1*, and *Hif1α*.

**Conclusions:**

This work suggests a sex-specific role for microglial iron import gene *Slc11a2* in propagating behavioral and cognitive phenotypes in the *APP/PS1* model of AD. These data also highlight an association between loss of a DAM-like phenotype in microglia and cognitive deficits in *Slc11a2*^KD^ *APP/PS1* female mice. Overall, this work illuminates an iron-related pathway in microglia that may serve a protective role during disease and offers insight into mechanisms behind disease-related sex differences.

## Background

Alzheimer’s disease (AD) is one of the most common neurodegenerative diseases and the most frequent cause of dementia. The disease is primarily characterized by the accumulation of extracellular amyloid-beta (Aβ) plaques and intraneuronal neurofibrillary tau tangles, which is thought to lead to neuronal loss and debilitating impairments in memory and cognition (1). In addition to Aβ and tau, other pathological features have been shown to contribute to AD development, including significant neuroinflammation, synaptic dysfunction, oxidative stress, and lysosomal dysfunction (2, 3). Additionally, mounting evidence demonstrates that excessive iron deposition in the brain is strongly associated with AD pathogenesis (4–6). Iron levels in the brain increase significantly with age (7, 8) and studies in patients with AD demonstrate that the degree of iron load in disease-associated brain regions (i.e., the hippocampus and frontal cortex) positively correlates with aberrant protein aggregation and severity of cognitive decline (9–11). Furthermore, iron has been found in dense core plaques and tau tangles in the brains of AD patients and mouse models (12–14), and directly binds to and exacerbates the toxicity of Aβ (15, 16). Although iron is critical for myelination, neurotransmitter synthesis, and mitochondrial metabolism in the healthy brain, excessive levels of iron can result in the harmful formation of toxic free radicals and production of reactive oxygen species (ROS), which can ultimately lead to lipid peroxidation, cellular damage, and ultimately cell death (17).

Microglial cells are the primary resident innate immune cell of the central nervous system (CNS) and play essential roles in brain development, maintenance of neural homeostasis, and response to injury and disease in the CNS. While it was been widely appreciated that microglial-mediated neuroinflammation is a key pathological hallmark of AD (18, 19), more recent work has also highlighted the prominent role microglia play in mediating brain iron dysregulation in disease (20–22). Microglia are equipped with the necessary machinery to import, store, and export and/or recycle iron (20, 23, 24). In fact, iron transport may occur preferentially in microglia compared to other cell types in the brain (25–27). Despite their high capacity to handle and store iron, microglia are particularly susceptible to iron-induced damage (28) and Ryan et al. recently demonstrated a predominant role for microglia in mediating the harmful effects of excess iron on other neural cells in a tri-culture system (29). Microglia are loaded with iron in AD and other neurodegenerative diseases, (30–34) and one of the key transcriptional changes in clusters of disease-associated microglia (DAMs) is an alteration in iron-storage genes such as *Fth1* and *Ftl* in both humans and mice (35, 36). While microglial iron loading has been more widely recognized as a key component of AD pathology, it is still not understood how microglial iron-handling contributes to overall disease progression (5, 37, 38).

At the cellular level, an intimate relationship between microglial iron load and inflammatory signaling has been established. In a reciprocal manner, iron can enhance markers of inflammation and oxidative stress in some systems (21, 39, 40), and inflammatory signals induce the cellular uptake and storage of iron (24, 41). Specifically, microglia preferentially upregulate iron importer divalent metal transporter 1 (DMT1; gene name, *Slc11a2*) in response to acute inflammatory stimuli such as lipopolysaccharide (LPS) and Aβ (24, 41, 42). DMT1 is a widely-expressed proton-coupled ferrous iron (Fe^2+^) importer essential for life, found on both the cellular plasma membrane and endosomal membrane (43). This importer plays a role in both transferrin-bound and non-transferrin-bound iron uptake, as it mediates the immediate import of ferrous iron at the cell surface, and also transports iron reduced in the endosome into the cytosol so it can be utilized by the cell (44). Previous work has targeted DMT1 in cell culture systems resulting in a significant decrease in pro-inflammatory IL1β signaling in response to an acute stimulus of Aβ (45). Furthermore, our work showed that knocking down *Slc11a2* in a short-term *in vivo* model of LPS-induced inflammation blunts the neural inflammatory response in male, but not female, mice (42). These results were observed in the absence of an additional iron load, suggesting a role for microglial DMT1 in helping to drive the baseline inflammatory response.

With these findings, it is intriguing to consider a role for microglial DMT1 in a disease of chronic cellular iron load and inflammation. However, to our knowledge, no studies have investigated whether targeting this microglial iron importer alters disease pathogenesis *in vivo*. In these studies, we generated an inducible, microglial-specific genetic knockdown of *Slc11a2* in a model of AD in both male and female mice. We investigated whether microglial *Slc11a2* knockdown alleviated markers of disease including microglial inflammatory and oxidative stress markers and changes in behavior and cognition.

## Materials and methods

### Experimental Animals

All mouse breeding, maintenance, and procedures were approved in advance and conducted in compliance with the Institutional Animal Care and Use Committee at Vanderbilt University. For the primary cell experiments from young and aged mice, young 9-week-old control C57BL/6J male mice were purchased from Jackson Laboratories (Bar Harbor, ME, USA) (#000664, JAX). C57BL/6J male mice between 27–30 months old were originally purchased from Jackson Laboratories and were aged and maintained in the Vanderbilt mouse facility. To determine the effect of decreased microglial DMT1 on disease, we generated a novel transgenic mouse model with inducible knockdown of *Slc11a2* in microglial cells in the *APP/PS1* model of AD. *Cx3cr1*^*Cre* − ERT2^ mice (B6.129P2(C)-*Cx3cr1*^*tm2.1(cre/ERT2)Jung*^/J; #020940) purchased from Jackson Laboratories (JAX, Bar Harbor, ME, USA) express a tamoxifen-inducible Cre-recombinase driven by the promoter for the microglial/macrophage *Cx3cr1* chemokine receptor gene, allowing for conditional knockdown of *loxP*-containing genes in *Cx3cr1*-expressing cells (46). *Slc11a2*-’*floxed*’ mice (129S-*Slc11a2*^*tm2Nca*^/*J*; #017789, JAX) (47) mice were bred with *Cx3cr1*^*Cre − ERT2*^ homozygous mice to obtain *Slc11a2*^*flfl*^;*Cx3cr1*^*Cre++*^ homozygous animals. *APP/PS1^+^* hemizygous animals were purchased from JAX and maintained in our facility (Tg(APPswe,PSEN1dE9)85Dbo; MMRRC_034832-JAX). These transgenic animals express a chimeric mouse/human amyloid precursor protein (Mo/HuAPP695swe) and a mutant presenilin-1 (PS1-dE9), and have been widely used in AD research, particularly in relation to amyloid-β associated pathology (48–50). We chose this model of AD based on the well-characterized development of disease-associated symptoms (i.e., amyloid deposition, cognitive deficits) and the progressive nature of disease development over the course of several months. This slower onset compared to other models allows us to examine the early pathological changes that occur prior to the onset of symptoms later in the course of disease. Additionally, the *APP/PS1* model has already been shown to exhibit significant microglial iron loading (21, 32), and an amyloid-driven model is relevant based on associations between iron and Aβ in the brain (15, 51). *APP/PS1^+^* hemizygous animals were bred separately with *Slc11a2*^*flfl*^ animals to yield *Slc11a2*^*flfl*^;*APP/PS1^+^* mice. Resulting progeny from these crosses were then bred with *Slc11a2*^*flfl*^;*Cx3cr1*^*Cre* − ERT2++^ animals to yield triple-transgenic *Slc11a2*^*flfl*^;*Cx3cr1*^Cre − ERT2+/−^;*APP/PS1^+^* or *APP/PS1*^−^ (i.e., ‘WT’) mice (**Additional File 1: Fig. S1A**). All mice used in experiments were *Slc11a2*^*flfl*^;*Cx3cr1*^Cre − ERT2+/−^ and either *APP/PS1^+^* hemizygotes or WT as littermate controls. Experimental mice were on a mixed 129S/BL6 background, with > 80% BL/6J genetic makeup. All genotypes were confirmed with an ear snip via Transnetyx (Cordova, TN) using real-time PCR. Mice were weaned at 3 weeks of age and had ad *libitum* access to food (*LabDiets*, standard rodent chow 5001, 240 ppm iron) and water. Both male and female mice were used in our experiments and were group-housed (2–5 per cage) by sex in transparent cages at 22–25°C under a 12 h light/dark cycle in a specific pathogen-free facility. Control and experimental animals were randomly assigned across cages.

### Tamoxifen Treatment

Tamoxifen (Sigma #T5648) was dissolved in corn oil (Sigma #C8267–2.5L, lot #MKCK6411, Saint Louis, MO) to generate a 20 mg/mL stock concentration by sonicating the mixture and stirring overnight in a glass vial at 37°C. *Slc11a2*^*flfl*^;*Cx3cr1*^*Cre* − ERT2+/−^;*APP/PS1^+^* ^*or* −^ male and female mice at 5–6 months of age were administered a dose of 4 mg (maximum 200 μL volume) tamoxifen via oral gavage every day for five consecutive days (42, 52) (**Additional File 1: Fig. S1B**). All mice that received tamoxifen are denoted as ‘*Slc11a2*^KD^’, and are either *APP/PS1* or WT. Littermate mice with the same genotypes (*Slc11a2*^*flfl*^;*Cx3cr1*^*Cre* − ERT2+/−^;*APP/PS1^+^* ^or −^) were administered gavage with corn oil as a control for the presence of Cre based on work showing effects of *Cx3cr1*-CreERT2 genotype alone on microglial function (53, 54). Corn-oil-treated animals are denoted as ‘Control,’ and are either *APP/PS1 + or* WT. The numbers of experimental animals used are shown in Supplemental Table 1. We chose to induce knockdown of *Slc11a2* between 5–6 months of age in these mice, as it is a relatively early timepoint in this AD model when Aβ plaque deposition becomes visible and allowed us to assess the effect of early changes in microglial *Slc11a2* on downstream disease development. Knockdown of *Slc11a2* was confirmed in isolated microglia from all animals via RT-qPCR utilizing a primer targeting *Slc11a2* exons 6–8 (**Additional File 1: Fig. S1C**).

### Behavioral assays

All behavioral assays were conducted in the Vanderbilt Murine Neurobehavioral Core after mice were acclimated to the facility for at least one to two weeks. All mice underwent testing by two experimenters between 12–15 months of age (mouse numbers and weights shown in **Additional File 2: Table S1, S2**). The running order of assays was kept consistent for all animals in each study, and animals were run each day between 0630–1300 h with one task per day. For each task, mice were acclimated to the testing room for 30 min to 1 h prior to testing, and control and experimental groups were evenly and randomly distributed across cages, days, and time of each assay. Following completion of a trial, each apparatus was cleaned of feces, disinfected, and deodorized with an anti-bacterial spray (Peroxigard, Virox Technologies) in between animals. *APP/PS1^+^* mice are known to be prone to spontaneous seizures (55) and any mouse that exhibited a seizure during an assay was excluded from that analysis (*n* = 3 male *Slc11a2*^KD^;*APP/PS1*^+^).

### Nest building

As a measurement of general cognition and well-being, an overnight nest building assay was used. Nest building assessments were performed as the first behavioral task to minimize effects of stress on the mice from other behavioral assays. Mice were single-housed and given 5 g of cotton nestlet (Ancare, Bellmore, NY) in the afternoon the day prior. The next morning, amount shredded and quality of nests was scored by a blinded observer using a 0–5 scale adapted from previous work, in 0.5 increments (42, 56, 57). Following nest building assessment, mice were re-housed in groups of 4–5 before all other behavioral tasks.

### Locomotor activity: Elevated Zero Maze and Open Field

For locomotor activity assessment, several assays were used. An elevated zero maze (white maze, width 5 cm; diameter 50 cm; wall height 15 cm, Stoelting Co. IL) was used first, where mice underwent a single 5 min trial of free exploration. Mice were video-recorded using a ceiling-mounted camera and movement was automatically tracked and scored using AnyMaze (Stoelting Co., Wood Dale, IL). Analysis parameters were set to ensure 80% of the mouse needed to be present in either the ‘open’ or ‘closed’ zone for an entry into that zone to be recorded. Total time in the open and closed zones and total distance traveled were measured. Sound-attenuating transparent open field chambers (27.5 × 27.5 cm) were used for a second measurement of baseline locomotor activity. Mice were placed in the center of the chamber and allowed to explore freely for 45 min. Distance traveled was recorded automatically via the breaking of infrared beams (MedAssociates ENV-510 software, Fairfax, VT). Additionally, time spent in the center area (19.05 × 19.05 cm) versus time in the ‘surround’ was calculated as a control measure of anxiety-like behavior.

### Short-term spatial working memory

A single-trial Y-maze was used as another measurement of baseline locomotor and exploratory behavior, as well as an assay to measure short-term working memory function. A clear plexiglass three-arm Y-maze (each arm 5 cm in width, 34.5 cm long) with differentiated arms (different colors of paper with or without patterns placed underneath the maze) was used. All mice were placed in the same point of the same arm and allowed to freely explore for 6 min. A ceiling-mounted camera recorded video of the mice and AnyMaze automatically measured total distance traveled and order of arm entries. Entry into another arm was predicated on having at least 80% of the mouse cross into at least 1 cm of the arm. Spontaneous alternation as a measure of intact working memory was calculated by hand using arm entry order data from AnyMaze. A ‘correct’ alternation is defined by three consecutive entries into three different arms (e.g., ABC, BCA, CAB). Percent alternation was calculated using: ((Number of spontaneous alternations) / (Number of total arm entries – 2)) * 100.

### Morris water maze

Mice underwent testing in the Morris water maze (MWM) to assess the effect of *Slc11a2* knockdown on learning and memory (58). Briefly, a circular pool approximately 1 m in diameter filled approximately 30 cm deep with 22–27°C water was used for this task. A white round platform (10 cm in diameter) was used to provide animals an escape from the water. Mice first underwent two visual training days, where the platform jutted above the water with a pole attached to allow mice to see the target platform. This platform was moved around to each of the four quadrants on each session during training days to allow the opportunity for each animal to swim and survey the room, which contained multiple visual spatial cues kept constant throughout. Each training day comprised four trials per mouse, and each mouse was given 60 sec to find the platform. If a mouse did not reach the platform in 60 sec, it was guided to and placed on the platform for at least 5 sec. On subsequent days following the two visual training days, the water was made opaque with non-toxic tempura white paint, and the platform was submerged approximately 0.5 cm under the water. The platform was kept in the same location for each trial and day, and mice were randomly placed in different locations in the pool so that the use of spatial cues for navigation was necessitated. Mice underwent four trials per day for five days, with each trial lasting 60 sec to assess learning and short-term memory. If mice did not find the platform within 60 sec, they were guided to the platform and escape latency was recorded as 60 sec. Following the final day of testing, the platform was removed and mice were allowed to swim freely for 60 sec. Total time spent in the target quadrant where the platform used to be, time spent around the location of the platform, swim speed, total distance traveled, and time spent in perimeter were recorded as measurements of platform location memory.

### Fear conditioning assay

Following completion of all other behavioral tasks, a fear conditioning assay was conducted to assess differences in fear-associated memory. Mice were placed in sound-attenuating chambers with a wire grid floor. On the first day (training trial), mice were placed in the chambers for 8 min and allowed to explore freely. Every 2 min, a 30 sec tone was played, followed immediately by a small shock administered through the wire floor (1 sec, 0.5 mA). This tone-shock pairing occurred 3 times during the training trial. To assess contextual fear conditioning, mice were placed back into the same chamber the next day and allowed to run around freely for 4 min with no tone or shock presented. Total time freezing – indicative of fear memory – was recorded automatically (VideoFreeze, MedAssociates). To assess cued fear conditioning (memory of the tone), mice underwent a second testing trial. This trial included a different experimenter handling the mice, significant alterations to the chamber with white walls, white floor inserts, and red light, and the scent of vanilla placed in an open tube outside the chamber. Mice freely explored the chamber for 2 min before the tone was administered for the final 2 min (without a shock pairing). Total time spent freezing during the no-tone and tone segments were recorded as a measurement of cued fear memory.

### Mouse euthanasia and tissue collection

At the time of euthanasia, mice were deeply anesthetized with isoflurane and 500–700 μL of blood was collected via cardiac puncture. Immediately following blood collection, mice were trans-cardially perfused with 20 mL of cold 1x Dulbecco’s phosphate-buffered saline (DPBS) to remove circulating blood and decapitated for rapid brain removal. Whole brains were either placed on ice for mincing and processing for cellular isolation, or bilateral hippocampus was isolated first before proceeding to cellular isolation.

### Tissue digestion and single-cell suspension preparation

Brains were rapidly removed and Briefly placed in 3 mL cold, sterile 1x Hank’s buffered saline solution (HBSS, Gibco, #14175095) containing 1% fetal bovine serum (FBS, heat-inactivated; Gibco, #10082147) to remove any residual blood. Microglia isolation was performed following published protocols, with slight modifications (59–61). Briefly, whole brains were transferred and finely minced with scissors in cold, sterile “IMG media” [Dulbecco’s modified Eagle’s medium (DMEM) with high glucose (4.5 g/L) and L-glutamine media (Gibco, #11965092) containing 10% FBS and 1% penicillin-streptomycin (Gibco, #15140122). Minced tissue was transferred into 50 mL conical tubes and 5 mL of digestion media (IMG media + 100 units Papain, #LK003176; 500 Kunitz units DNase, #LK003170, Worthington Biochemicals, Lakewood, NJ) was added to each tube. Whole-brain samples were enzymatically-dissociated by placing in an orbital shaker for 1 h at 37°C, diluted with 10 mL IMG media, and strained through 70 μm sterile filters (Corning, #431751). Bilateral hippocampus samples in the RNA-sequencing studies were treated in the same manner described above, with slight modification. Bilateral hippocampus samples were isolated and immediately placed in 15 mL conical tubes on ice and 2.5 mL digestion media was added. Samples were incubated for 30 min in an orbital shaker at 37°C. Every 10 min, samples were triturated up and down with a serological pipette of decreasing size before straining samples through filters and proceeding with subsequent steps. All samples were further processed at 4°C unless otherwise indicated.

### Percoll gradient

Cells were centrifuged for 5 min at 500 × g, re-suspended in a solution of 30% isotonic Percoll and IMG media (Cytiva, #17-0891-01), and slowly layered onto a 70% Percoll gradient with HBSS + 1% FBS. HBSS + 1% FBS (without Percoll) was layered on top, and samples were centrifuged for 15 min at room temperature at 600 × g with the brake set to the lowest setting to allow for density separation. The supernatant containing myelin and neuronal debris was removed, and cells at the interface between the 30–70% gradients were carefully collected and placed on ice into 8 mL HBSS + 1% FBS in a fresh tube to wash residual Percoll. Cells were centrifuged at 500 × g for 5 min at 4°C, and pelleted cells were re-suspended in appropriate media for downstream assays.

### Plating and treatment for primary cell experiments

For experiments conducted in isolated primary glial cells from young and aged mice, all steps above were performed under sterile conditions in a cell culture hood with autoclaved tools and sterile-filtered reagents. Glial cells isolated and pelleted from the Percoll gradient were re-suspended in 1 mL IMG media for counting and plating. Cells were counted using the Nexcelom Cellometer Auto T4 Cell Counter (Nexcelom Biosciences) and plated at a density of 100,000 cells per well in poly-L-lysine-coated 48-well plates in pre-warmed sterile IMG media containing 5 ng/mL GM-CSF (R&D Systems, #415-ML-010). Media was changed the next day, and then every other day for five days before stimulation with Aβ as described below.

### CD11b immunomagnetic microglial isolation

For experiments analyzing gene expression (RNA sequencing and RT-qPCR) in microglia, Percoll-isolated glial samples were further processed for enrichment of CD11b^+^ microglia. Following Percoll gradient separation, centrifugation, and pelleting, cells were re-suspended in 400 μL cold “MACS” buffer (1x PBS containing 0.5% FBS and 2 mM EDTA) and transferred to 5 mL tubes. Cells were centrifuged at 4°C for 5 min at 500 × g, pelleted, and re-suspended in 90 μL MACS buffer for magnetic labeling and separation according to manufacturer’s instructions (Miltenyi Biotec, Bergisch Gladbach, Germany). Briefly, samples were incubated with magnetic anti-CD11b MicroBeads (Miltenyi Biotec, #130-093-634; 10 μl per 90 μl buffer/brain) for 15 min at 4°C. Magnetic separation was performed utilizing MS columns, and CD11b^+^ cells and the effluent non-magnetic fractions (CD11b^−^ cells) were obtained. Following a final centrifugation for 5 min at 500 × g, cells were immediately re-suspended in RLT lysis buffer (Qiagen, #74004) supplemented with 1% beta-mercaptoethanol, Briefly vortexed, and flash-frozen in liquid nitrogen. Samples were stored at −80°C until RNA isolation.

#### In vitro cells and experimental treatments

The immortalized microglial cell line, “IMG” (62), was used for *in vitro* experiments to assess the effect of pharmacological inhibition of DMT1 on Aβ-induced inflammation. IMG cells were purchased from Millipore (Cat. #SCC134, RRID:CVCL_HC49), and cultured as described using Accutase for dissociation and passaging (45). Briefly, cells were cultured up to a maximum of 10 passages in sterile Dulbecco’s modified Eagle’s medium (DMEM) with high glucose (4.5 g/L) + 2.5 mM glutamine (Gibco, #11965092) supplemented with 10% fetal bovine serum (FBS, heat-inactivated, Gibco, #16140071) and 1% penicillin/streptomycin (“IMG media”).

### Ebselen treatments

The drug ebselen [2-phenyl-1,2-benzisoselenazol-3(2*H*)-one] was chosen as a robust inhibitor of DMT1 (63). Ebselen was purchased from Focus Biomolecules (#10–2288) and re-suspended in sterile dimethyl sulfoxide (DMSO; Sigma, #276855). IMG cells were plated and grown overnight in six-well-plates (150,000–200,000 cells/well) in IMG media. The next day, cells were treated for 24 h with either 25 μM ebselen or control DMSO. This concentration of ebselen was chosen as the treatment dose following preliminary experiments indicating this dose decreased cellular iron content and following similar reported doses from previous work (64). Following 24 h of ebselen/DMSO treatment, cells were further treated as described below.

### Amyloid-β and iron treatments

In both IMG cells and primary isolated glia in the young and aged mice experiments, amyloid-β_1–42_ was used as an acute AD-associated inflammatory stimulus. Aβ (HFIP-treated, rPeptide #A-1163–2) and scrambled Aβ (rPeptide #A-1004–2) were purchased from rPeptide and 5 mM stock solutions were prepared with sterile, anhydrous DMSO (Sigma #276855) and sonicated for 15 min before storing aliquots at −20°C. The day before cell stimulation, oligomeric Aβ_1–42_ was prepared as previously described (39) using cold, sterile phenol-free Ham’s F-12 media (R&D Systems, #M25350) and allowed to rest at 4°C for 24 h. The next day, cells were treated with 1 μM Aβ_1–42_ or scrambled Aβ for 24 h before lysis and collection for RNA isolation. For *in vitro* experiments in IMG cells, ferric ammonium citrate (FAC, Sigma, #F5879) was used as a non-transferrin-bound form of iron. FAC was re-suspended fresh in sterile RNase-free water immediately before each experiment, and cells were treated for 24 h with 50 μM FAC based on literature recommendations (39, 65) or water (control), with or without Aβ prior to lysis and collection for RNA isolation or ICP-MS, as described below.

### Inductively-coupled Plasma Mass Spectrometry (ICP-MS)

Following 24 h of treatment with scrambled Aβ or 1 μm Aβ_1–42_ ± FAC, IMG cells were collected for ICP-MS analysis of intracellular iron content. After washing twice with ice-cold 1x PBS, cells were collected into metal-free tubes using Accutase, and total cell counts were measured for data normalization. After centrifugation at 600×g for 5 min and removal of supernatant, cells were acid-digested in 150 μL trace-metal grade nitric acid (70%, OPTIMA Grade HNO_3,_ Fisher-Sci, #A467–250), and 30% ultra trace-grade hydrogen peroxide (Thermofisher) was added at a 1:4 dilution (37.5 μL H_2_O_2_). Samples were vortexed, incubated at 65°C overnight, and diluted the next day with Ultrapure Milli-Q water (Ω18.2) at 10 times the volume of nitric acid (1.5 mL water). ICP-MS was performed at the Vanderbilt Mass Spectrometry Research Center using an Agilent 7700 ICP-MS (Agilent) attached to a Teledyne autosampler (CETAC Technologies, Omaha, NE). The following settings were used: cell entrance = −40V, cell exit = −60V, plate bias = −60V, OctP bias = −18V, and collision and cell helium flow = 4.5 mL/min. Samples were introduced by peristaltic pump and taken up at 0.5 rps for 30 s, followed by 30 s at 0.1 rps for signal stabilization. A calibration curve for each isotope was made at 0, 1, 10, 100, 1000, 5000, and 10,000 ppb, and blanks were run following standard calibration to wash out signal from the 10,000 ppb standard. Data were acquired and analyzed using the Agilent Mass Hunter Workstation Software version A.01.02.

### RNA isolation, cDNA synthesis, and RT-qPCR

Lysed cell samples from all experiments (i.e., CD11b + microglia and primary isolated glia) were processed for total mRNA using an RNeasy Micro Kit with DNase treatment according to manufacturer’s instructions (Qiagen, Hilden, Germany, #74004), with the exception of the IMG experiments, which used the RNeasy Mini Kit (Qiagen, # 74104). Following on-column RNA purification and elution, cellular RNA was reverse transcribed into cDNA at equal concentrations across samples using iScript Reverse Transcriptase (BioRad, Hercules, CA). RT-qPCR was conducted to assess the expression of several genes and confirm *Slc11a2* knockdown using FAM-conjugated TaqMan Gene Expression Assay primers (Thermofisher, shown in **Additional File 3: Table S3**) and iQ Supermix (BioRad). PCR reactions were performed in duplicate under thermal conditions: 95°C for 10 min, followed by 40 cycles of 95°C for 15 s, and 60°C for 45 s. The expression of each gene measured was normalized to a housekeeping gene (either *18S* or *ActinB* where indicated), and relative expression values were analyzed utilizing the comparative cycle threshold 2^−ΔΔCT^ method (66).

### RNA sequencing and library preparation

Following on-column purification and DNase treatment with the Qiagen RNeasy Micro Kit, total mRNA extracted from hippocampal CD11b^+^ samples was submitted to the Vanderbilt Technologies for Advanced Genomics (VANTAGE) Core facility for sample quality control assessment and RNA sequencing (RNA-seq). Only hippocampal CD11b^+^ microglia isolated from female animals were used for RNA-seq, following earlier findings of significant behavioral differences primarily in *Slc11a2*^KD^ female *APP/PS1* animals. The concentration of RNA samples was determined by NanoDrop (ThermoScientific). Sample Quality Control analysis was assessed using fluorometry Qubit and integrity by BioAnalyzer, and a RIN value of > 7 was confirmed for all samples before proceeding to library preparation and sequencing. Paired-end sequencing libraries were constructed using a standard mRNA NEBNext Poly(A) selection Library Prep Kit (Illumina). Library Quality Control analysis was performed by using Qubit and BioAnalyzer to determine the concentration and size bp. Samples were then sequenced at multiplex Paired-End 150 bp using the Illumina NovaSeq 6000 sequencing platform. To confirm sequencing quality, Illumina Quality Scores were calculated utilizing the following equation: Q = −10log_10_I. All samples sequenced reached sequencing quality of at least Q(30).

### Sequencing analysis: alignment, mapping, quantification, differential expression

Gene alignment, read mapping, gene counts quantification, and differential gene expression analyses were conducted at the Creative Data Solutions (CDS) Core at Vanderbilt. RNA-seq reads were adapter-trimmed and quality-filtered using Trimgalore v0.6.7 (67) and Cutadapt 1.18 (68) to remove adapter sequences and pairs that were either shorter than 20 bp or that had Phred scores less than 20. An alignment reference was generated from the mm39 mouse genome and GENCODE comprehensive gene annotations (M31), to which trimmed reads were aligned and counted using Spliced Transcripts Alignment to a Reference (STAR) v2.7.9a (69) with the –quantMode GeneCounts parameter. About 30–50 million uniquely mapped reads were acquired per sample. DESeq2 package v1.36.0 (70) was used to perform sample-level quality control, low count filtering, normalization and downstream differential gene expression analysis. Genomic features counted fewer than five times across at least three samples were removed. The measure of standard deviation (sd) and quantiles on principal component 1 (PC1) among samples was used to assess whether any samples were a statistical outlier. One sample in the Control *APP/PS1* group was removed from analyses after exhibiting a deviation of > 2 standard deviations and an interquartile range of > 1.5 in PC1 compared to its respective group (sample shown in **Additional File 4: Fig. S2B and C**). Five to six biological replicates per condition were included for the differential expression analysis. Differentially expressed genes were identified using a false discovery rate (FDR) adjusted p-value threshold of 0.05, calculated using the Benjamini-Hochberg (BH) procedure for multiple hypothesis testing correction, and a log2 fold change threshold of greater than 1. Gene set enrichment analysis (GSEA) (71) was performed using the R package Clusterprofiler (72) with gene sets from the Mouse MSigDB database (73). Coverage of reads across annotated exons in the *Slc11a2* gene analysis was done using the R package ggcoverage 1.3.0 (74). All data processing was performed at the Advanced Computing Center for Research and Education (ACCRE) at Vanderbilt University.

### Data and statistical analyses

Data are presented as mean ± S.E.M. All experiments were analyzed using analysis of variance (ANOVA) for multiple comparisons followed by appropriate post-hoc analyses unless otherwise noted. Male and female data were first compared using ANOVA (2(Sex) × 2(Genotype) × 2(Treatment), followed by Sidak’s corrections for multiple comparisons and analysis of interaction effects. Based on our previous work showing sex differences in *Slc11a2* expression between males and females, most primary analyses were conducted within each sex separately to assess the effect of *Slc11a2* knockdown in each sex. To do this, a 2(Genotype) × 2(Treatment) ANOVA followed by Sidak’s corrections was used. In analyzing MWM data, repeated measures ANOVA (2(Knockdown) × 2(*APP/PS1* Genotype) × 5(Day)) was used to analyze latency data from multiple training days and Tukey’s post-hoc analysis was used following significant F values to establish differences among all groups. Data from primary cell and IMG cell experiments were analyzed using either 2(Treatment) × 2(Age) ANOVA or 3(Treatment) × 2(ebselen/DMSO) ANOVA, respectively. Sidak’s post-hoc analysis was used for interaction effects and corrections for multiple comparisons. Statistical outliers within each group for all studies were identified using either the ROUT or Grubb’s method for outliers and excluded from statistical analyses. GraphPad Prism 9 (GraphPad Software, San Diego, CA, USA) was used for statistical analyses outside of RNA-seq analyses conducted in R. Differences among groups were considered significant at values of p < 0.05.

## Results

### Age and Aβ stimulation synergize to increase microglial Slc11a2 and iron loading markers in primary microglia.

**Figure 1:**
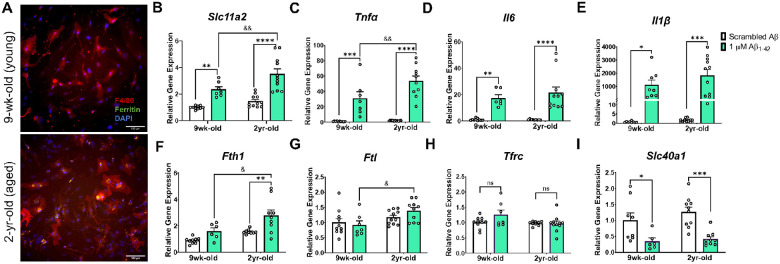
Age and Aβ stimulation synergize to increase microglial *Slc11a2* and iron-loading markers in primary microglia. **A)** Representative images of Percoll-isolated glia from young (top image, 9-week-old) and aged (bottom image, 2-year-old) mouse showing ferritin deposits in microglia from the aged mouse. Isolated glia were stained with antibodies raised against ferritin-L and F4/80, along with DAPI to visualize ferritin, microglia, and nuclei, respectively. Images shown at 20x, scale bar = 100 μm. **B-I)** Relative gene expression (compared to control scrambled Aβ) of **B** *Slc11a2*, **C** *Tnfα*, **D** *Il6*, **E** *Il1β*, **F** *Fth1*, **G** *Ftl*, **H** *Tfrc*, and **I** *Slc40a1* via RT-qPCR. Isolated cells from young and aged mice were plated and treated with scrambled Aβ or 1μM Aβ_1–42_ for 24 h before collection for RNA isolation and RT-qPCR analysis. Two-way ANOVA, *p<0.05, **p<0.01, ***p<0.001, ****p<0.0001 effect of treatment. ^&^p<0.05, ^&&^p<0.01 effect of age × treatment. ns = not significant. Data represent the mean ± S.E.M of 7–11 mice per group. Statistical outliers were removed using the Grubb’s test.

To assess a potential role for microglial iron and *Slc11a2* in aging and amyloid-related pathology, we isolated glia from young and aged mice for primary cell *in vitro* experiments. We first observed significant ferritin (FtL) protein deposits in cells isolated from aged compared to young mice (Fig. 1A), demonstrating, as others have shown, a key iron-loading microglial phenotype in aging (38, 75). To determine whether *Slc11a2* contributes to this age-associated increase in iron and whether the transporter gene plays a role in amyloid-related disease conditions, isolated microglia from young and aged mice were treated *in vitro* with an acute stimulus of 1 μM oligomeric Aβ for 24 h and gene expression of *Slc11a2* was measured. As others have also shown (24, 45), there was a significant increase in microglial *Slc11a2* in response to acute Aβ exposure (Fig. 1B, *Treatment*, F(1, 35) = 48.91, p < 0.0001). Additionally, glia from the two-year-old aged mice exhibited an augmented Aβ-induced *Slc11a2* response, which was significantly greater than the response observed in the cells from young mice (*Age*, F(1, 35) = 11.21, p = 0.002; young vs. old Aβ, p = 0.005). In addition, there was a robust increase in pro-inflammatory cytokines *Tnfα, Il1β*, and *Il6* in response to Aβ (Fig. 1C–E, *Il6*: *Treatment*, F(1, 32) = 41.20, p < 0.0001; *Il1β*: F(1, 34) = 24.23, p < 0.0001; *Tnfα*: F(1, 34) = 77.83, p < 0.0001), which was even greater in the glia from the aged mice compared to those isolated from the young mice (significant for *Tnfα*: *Age*, F(1, 34) = 6.52, p = 0.015, *Interaction* F(1, 34) = 5.57, p = 0.024; young vs. aged Aβ p = 0.005). Along with differences in Aβ-induced *Slc11a2* gene levels in the glia from the aged mice, there was a significant increase in iron-storage genes *Ftl* and *Fth1* in response to Aβ only in the cells from the aged animals (Fig. 1F–G). Specifically, Aβ induced an increase in *Fth1* in the aged glia (*Age*, F(1, 29) = 12.46, p = 0.001, *Treatment*, F(1, 29) = 13.67, p = 0.0009), and *Fth1* and *Ftl* were significantly higher in response to Aβ in the aged cells when compared to the young cells (*Fth1*, young vs. aged, p = 0.01; *Ftl*: *Age*, F(1, 34) = 7.92, p = 0.008, young vs. aged, p = 0.02). There were no differences in *Tfrc* gene expression – another main iron importer gene – due to age or Aβ treatment (Fig. 1H, p > 0.05), suggesting that a specific gene expression increase in *Slc11a2* may accompany age- and Aβ-related changes in cellular iron and inflammatory status. Aβ also decreased *Slc40a1* levels (gene for ferroportin, main iron exporter) to a similar degree in the cells from the young and aged animals (Fig. 1I, *Treatment*, F(1, 30) = 23.40, p < 0.0001), further suggesting that a specific alteration in *Slc11a2* in response to age and amyloid may be involved in the progression of disease.

### DMT1 inhibition in vitro significantly blunts Aβ-induced inflammation and decreases cellular iron levels in immortalized microglia.

**Figure 2:**
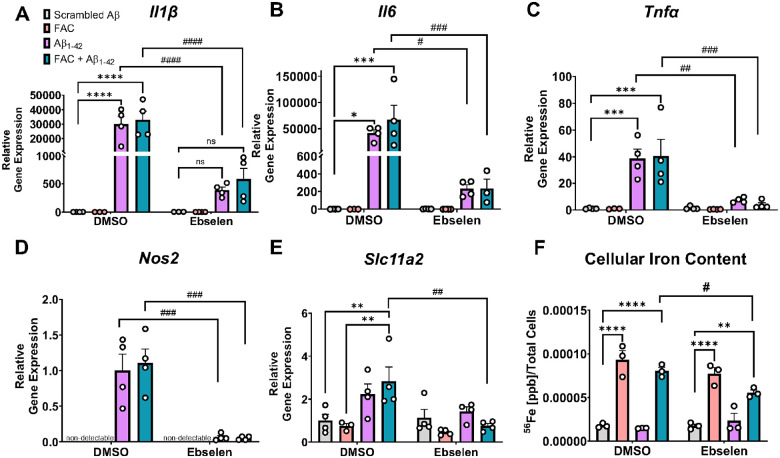
DMT1 inhibition *in vitro* significantly blunts Aβ-induced inflammation and decreases cellular iron levels in immortalized microglia. **A-E)** Relative gene expression (compared to scrambled Aβ DMSO) via RT-qPCR of **A** *Il1β*, **B** *Il6*, **C** *Tnfα*, **D** *Nos2*, and **E** *Slc11a2* in IMG cells. IMG cells were treated for 24 h with DMSO or 25 μM ebselen, followed by 24 h treatment with scrambled Aβ or 1 μM Aβ1–42 ± 50 μM ferric ammonium citrate (FAC). **F)** ICP-MS analysis of intracellular ^56^Fe content from IMG cells following 24 h treatment with DMSO or ebselen, and 24 h scrambled Aβ ± FAC or Aβ_1–42_ ± FAC treatment. Two-way ANOVA, *p<0.05, **p<0.01, ***p<0.001, ****p<0.0001 effect of Aβ or FAC treatment. ^#^p<0.05, ^##^p<0.01, ^###^p<0.001, ^####^p<0.0001 effect of treatment × ebselen. ns = not significant. Data show a representative experiment with the mean ± S.E.M of 3–4 technical replicates, and experiment was repeated three times. Statistical outliers were removed using the Grubb’s test.

Based on the purported roles for DMT1 in Aβ stimulation and iron load, we characterized the effect of inhibiting DMT1 on Aβ and iron-induced inflammation in an *in vitro* system. Cells from the murine immortalized microglial cell line, “IMG” cells (62), were treated with ebselen, a pharmacological inhibitor of DMT1 (63), before subsequent treatment with scrambled Aβ, oligomeric Aβ_1–42_ alone, or iron (50 μM FAC) + Aβ_1–42_. Aβ stimulation leads to a robust increase in microglial pro-inflammatory *Il1β, Il6, Tnfα*, and *Nos2* transcription, as expected (Fig. 2A–D, *Il1β*: *Treatment*, F(3, 22) = 16.78, p < 0.0001; *Il6*: *Treatment*, F(3, 23) = 5.28, p = 0.006; *Tnfα*: *Treatment*, F(3, 23) = 10.89, p = 0.0001; *Nos2*: *Treatment*, F(3, 23) = 16.31, p < 0.0001). Addition of 50 μM FAC did not have a significant effect on Aβ-induced inflammatory markers. Ebselen profoundly decreased the Aβ-induced pro-inflammatory cytokine response for all three cytokines assayed along with *Nos2*, even in the absence of excess iron added to the media (Aβ alone condition) (Fig. 2A–D, *Il1β*: *Interaction*, F(3, 22) = 15.76, p < 0.0001; *Il6*: *Interaction*, F(3, 23) = 4.81, p = 0.0096; *Tnfα*: *Interaction*, F(3, 23) = 6.89, p = 0.0018; *Nos2*: *Interaction*, F(3, 23) = 13.18, p < 0.0001). Aβ induced a significant upregulation in *Slc11a2* and ebselen inhibited this increase when a bolus of FAC was added as well (Fig. 2E, *Treatment*, F(3, 23) = 5.07, p = 0.008, *Interaction*, F(3, 23) = 3.49, p = 0.032). This was paralleled by a change in total intracellular iron levels as measured via ICP-MS, where ebselen significantly decreased cellular iron levels in the FAC + Aβ_1–42_ condition (Fig. 2F, *Treatment*, F(3, 16) = 72.53, p < 0.0001, *Ebselen*, F(1, 16) = 4.15, p = 0.058, *Interaction*, F(3, 16) = 3.52, p < 0.05). These data demonstrate associations between DMT1 inhibition, decreases in cellular iron levels, and blunted Aβ-induced pro-inflammatory responses in IMG cells.

### Microglial *Slc11a2* knockdown results in a hyperactive phenotype in female *APP/PS1* mice and worsens hyperactivity in male *APP/PS1* mice at 12–15 months.

To determine the effects of knocking down *Slc11a2* in vivo, we generated a transgenic mouse line allowing for inducible knockdown of *Slc11a2* in microglia between 5–6 months of age. Between 7–9 months after tamoxifen treatment, when mice were 12–15 months of age, male and female control WT, control *APP/PS1*, *Slc11a2*^KD^ WT, and *Slc11a2*^KD^ *APP/PS1* mice were run through a series of behavioral assays to assess the effect of microglial *Slc11a2* knockdown on aspects of behavior and cognition.

**Figure 3:**
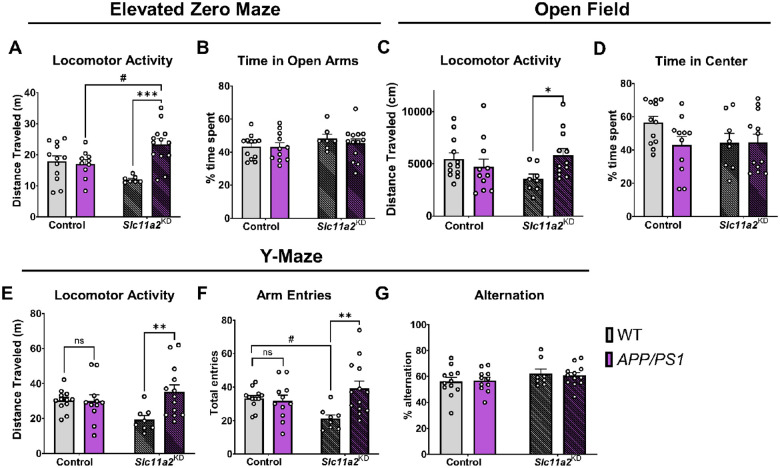
Microglial *Slc11a2* knockdown results in a hyperactive phenotype in female *APP/PS1* mice at 12–15 months. **A-B)** Elevated zero maze. **A** Total distance traveled (m) in control WT, control *APP/PS1*, *Slc11a2*^KD^ WT, and *Slc11a2*^KD^ *APP/PS1* female mice. **B** Total percent time spent in open arms. **C-D)** Open field locomotor activity assay. **C** Total distance traveled (cm). **D** Total percent time spent in the center. **E-G)** Exploratory Y-maze. **E** Total distance traveled (m). **F** Total number of different arm entries. **G** Total percent alternation. Two-way ANOVA, *p<0.05, **p<0.01, ***p<0.001 effect of *APP/PS1* genotype, #p<0.05 *Slc11a2*^KD^ vs. Control. ns = not significant. Data represent the mean ± S.E.M of 8–13 female mice per group. Statistical outliers were removed using the Grubb’s test.

First, to assess locomotor activity, mice were tested in elevated zero maze (EZ maze, 5 min), open field chambers (45 min), and one-trial spontaneous alternation Y-maze tests (6 min) and total distance traveled was measured in each. In females, control *APP/PS1* mice did not exhibit differences in baseline locomotor activity compared to control WT female mice in any of the assays tested (Fig. 3A–F; p > 0.05). However, microglial *Slc11a2*^KD^ female *APP/PS1* animals exhibited a significant increase in distance traveled in all three activity measurement assays compared to their non-*APP/PS1* counterparts (Fig. 3A, C, E, F; activity measurements, EZ maze: *APP/PS1*, F(1, 38) = 9.28, p = 0.004, *Interaction effect*, F(1, 38) = 12.29, p = 0.001; open field: *Interaction*, F(1, 39) = 5.36, p = 0.03; Y-maze activity: *APP/PS1*, F(1, 40) = 5.23, p = 0.03, *Interaction*, F(1, 40) = 5.92, p = 0.02; arm entries in Y-maze: *APP/PS1*, F(1, 40) = 5.76, p = 0.02, *Interaction*, F(1, 40) = 7.93, p = 0.008). As control measurements to assess for anxiety-like behavior, the amount of time spent in the open arms of the EZ maze (Fig. 3B, p > 0.05) or in the center area of the open field chambers were not significantly different (Fig. 3D, p > 0.05). Additionally, there were no significant differences in Y-maze spontaneous alternation capacity between any groups (Fig. 3G, p > 0.05).

**Figure 4:**
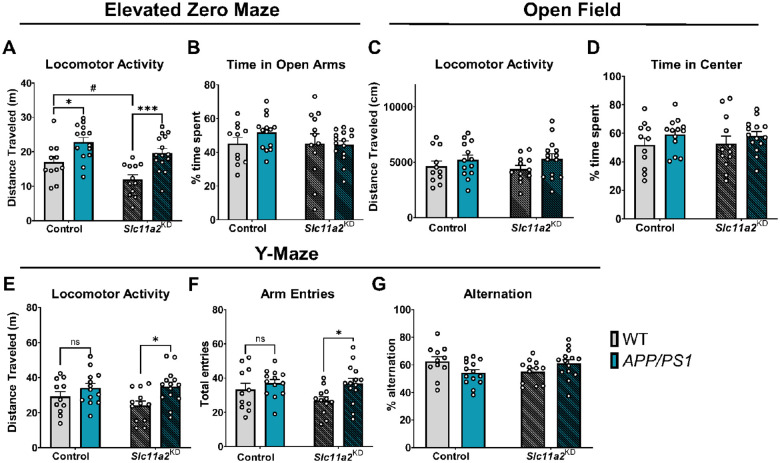
Microglial *Slc11a2* knockdown worsens hyperactivity in a novel environment in male *APP/PS1* mice at 12–15 months. **A-B)** Elevated zero maze. **A** Total distance traveled (m) in control WT, control *APP/PS1*, *Slc11a2*^KD^ WT, and *Slc11a2*^KD^ *APP/PS1* male mice. **B** Total percent time spent in open arms. **C-D)** Open field locomotor activity assay. **C** Total distance traveled (cm). **D** Total percent time spent in the center. **E-G)** Exploratory Y-maze. **E** Total distance traveled (m). **F** Total number of different arm entries. **G** Total percent alternation. Two-way ANOVA, *p<0.05, ***p<0.001 effect of *APP/PS1* genotype. ^#^p<0.05 *Slc11a2*^KD^ vs. Control. ns = not significant. Data represent the mean ± S.E.M of 11–15 male mice per group. Statistical outliers were removed using the Grubb’s test.

Male *APP/PS1* mice exhibited a significant increase in activity in the EZ maze compared to WT controls (Fig. 4A; EZ Maze: *APP/PS1 effect*, F(1, 48) = 22.61, p < 0.0001). There was a significant main effect of *Slc11a2* knockdown on activity in the EZ maze in males (*Knockdown effect*, F(1, 48) = 8.18, p = 0.0063), and post-hoc analyses revealed that *Slc11a2* knockdown had a greater effect on the hyperactive phenotypes observed in the *APP/PS1* males compared to corresponding controls (Fig. 4A, EZ maze: Control vs. *APP/PS1*, p = 0.013, *Slc11a2*^KD^ Control vs. *Slc11a2*^KD^ *APP/PS1*, p = 0.0006). This was observed in the absence of a significant anxiety-like phenotype, with no difference in time spent in the open arms of the EZ maze (Fig. 4B). Male *APP/PS1* mice did not show any significant differences in total distance traveled or anxiety-like behavior in the open field chambers over 45 min (Fig. 4C–D, p > 0.05).

However, there was a significant *APP/PS1*-associated increase in activity in a 6 min Y-maze in the males (Fig. 4E; Y-maze: *APP/PS1 effect*, F(1, 46) = 8.40, p = 0.006), which was exacerbated in the *Slc11a2* knockdown animals (Fig. 4E, Y-maze activity post-hoc comparisons: Control vs. *APP/PS1*, p = 0.39, *Slc11a2*^KD^ Control vs. *Slc11a2*^KD^ *APP/PS1*, p = 0.012; Fig. 4F, Y-maze arm entries: F(1, 46) = 5.65, p = 0.02; post-hoc comparisons: Control WT vs. *APP/PS1*, p = 0.61, *Slc11a2*^KD^ WT vs. *Slc11a2*^KD^ *APP/PS1*, p = 0.03). There were no significant differences in Y-maze spontaneous alternation capacity as a measure of working memory (Fig. 4G). Overall, these data suggest that microglial *Slc11a2* knockdown is associated with an exaggerated hyperactive phenotype in the *APP/PS1* animals, particularly in female mice.

### *Slc11a2* knockdown worsens memory performance in Morris water maze and cued fear conditioning assay in *APP/PS1* females.

**Figure 5:**
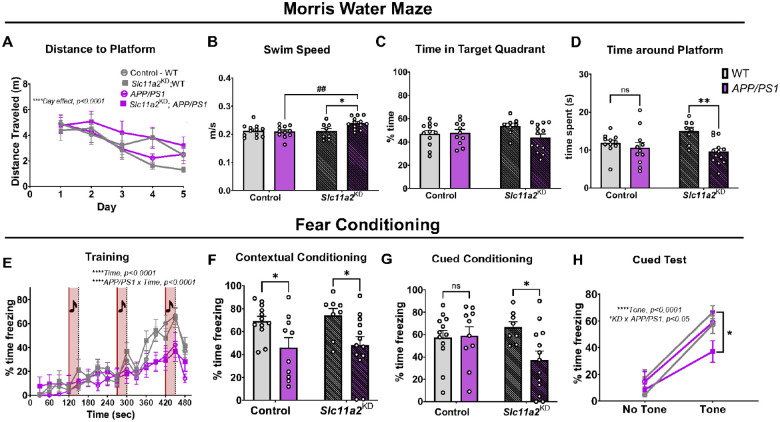
Microglial *Slc11a2* knockdown worsens memory performance in Morris Water Maze and cued fear conditioning assay in *APP/PS1* female mice. **A-D)** Morris water maze (MWM). **A** Total distance traveled (m) before reaching hidden platform over course of five training days in control WT, control *APP/PS1*, *Slc11a2*^KD^ WT, and *Slc11a2*^KD^ *APP/PS1* female mice. Four trials of 60 s each were conducted each day and averaged per animal. Three-way ANOVA, ****p<0.0001 effect of day. **B** Average speed (m/s) measured during probe trial. Two-way ANOVA, *p<0.05 effect of *APP/PS1* genotype. ^##^p<0.01 *Slc11a2*^KD^ vs. Control. **C** Total percent time spent in the target quadrant in probe trial for memory. **D** Total time (s) spent around where the platform previously was (exact platform location + 1.5 cm radius) during probe trial for memory. **E-H)** Fear conditioning assay. E Percent component time freezing during the 8 min training protocol. Every 2 min, a 30 s tone was played, followed by a mild foot-shock. Increased freezing behavior over the course of the assay is shown. ****p<0.0001 effect of time, ****p<0.0001 effect of *APP/PS1* × time. **F** Percent time freezing during 4 min contextual fear conditioning test. **G** Total percent time spent freezing during the 4 min of cued fear conditioning testing. **H** Percent component time spent freezing during 2 min of no-tone versus 2 min of tone presentation in cued fear conditioning test. ****p<0.0001 effect of tone, *p<0.05 *Slc11a2*^KD^ *APP/PS1* vs. Control *APP/PS1*. Data represent the mean ± S.E.M. of 8–13 mice per group. Statistical outliers were removed using the Grubb’s test.

To determine whether *Slc11a2* knockdown affected measurements of well-being, cognition, and longer-term learning and memory, several behavioral tasks were utilized. An overnight nest building assay revealed a robust *APP/PS1*-associated deficit in nestlet amount shredded in the females; however, there was no additional effect of *Slc11a2* knockdown on this measurement of cognition and well-being (Control WT mean, 4.3 g ± 0.28; Control *APP/PS1* mean, 1.73 g ± 0.30; *Slc11a2*^KD^ WT mean, 3.5 g ± 0.47; *Slc11a2*^KD^ *APP/PS1* mean, 1.48 g ± 0.35; *APP/PS1 effect*, F(1, 38) = 43.54, p < 0.0001). To assess learning and spatial memory, mice underwent five days of trials to find a hidden platform in Morris water maze (MWM), a widely used test for hippocampal-dependent spatial navigation and memory. Over the course of five days, all female mice (regardless of *APP/PS1* genotype or *Slc11a2* knockdown) effectively learned the location of the platform compared to their baseline on day one, exhibiting significantly shorter path lengths to find the platform by day five (Fig. 5A; *Day effect*, F(2.75, 110.1) = 11.38, p < 0.0001). Female *APP/PS1* mice were not different than control WT females at finding the hidden platform during training days. However, microglial *Slc11a2*^KD^ female *APP/PS1* animals exhibited slightly longer path lengths to find the hidden platform, although this was not statistically significant (p = 0.1). Furthermore, in accordance with data from earlier tasks assessing locomotor activity, female *Slc11a2*^KD^ *APP/PS1* mice were significantly more hyperactive in the water maze (i.e., greater average swim speed) compared to all other groups (Fig. 5B; Knockdown × *APP/PS1* Interaction, F(1, 40) = 5.45, p = 0.025). Mice underwent one 60 sec probe trial for memory of platform location 24 h after the last set of training trials, in which the platform was removed from the pool and mice were allowed to swim freely. There were no significant differences in time spent in the target quadrant where the platform location was previously (Fig. 5C, p > 0.05); however, female *APP/PS1* mice overall exhibited a decrease in time spent around the exact platform location (exact platform location, plus 1.5 cm surrounding radius) compared to WT littermate controls (Fig. 5D; Females: *APP/PS1 effect*, F(1, 39) = 8.90, p = 0.005). Female *Slc11a2*^KD^ *APP/PS1* mice exhibited a significant further reduction in time spent around the platform location, suggesting an exacerbated loss of memory function in these animals (Females: post hoc analysis: Control WT vs. Control *APP/PS1*, p = 0.68; *Slc11a2*^KD^ WT vs. *Slc11a2*^KD^ *APP/PS1*, p = 0.004). To further assess the effects of *Slc11a2* knockdown on memory function, we utilized a fear conditioning assay in which a tone was succeeded by a mild foot shock. During the initial training session, all groups significantly increased freezing by the third tone presentation, albeit *APP/PS1* females overall froze less over the course of the 8 min training session (Fig. 5E, *Time effect*, F(6.9, 279.3) = 41.1, p < 0.0001; *Interaction of Time* × *APP/PS1*, F(15,600) = 4.91, p < 0.0001). In the contextual fear conditioning assay, female *APP/PS1* mice exhibited a disease model-associated deficit in fear memory (Fig. 5F, *APP/PS1 effect*, F(1, 39) = 12.26, p = 0.0012); although, there was no additional effect of *Slc11a2* knockdown. However, in the cued fear conditioning memory task, female *Slc11a2*^KD^ *APP/PS1* mice displayed a significant worsening in fear memory associated with presentation of a tone (Fig. 5G, *Knockdown* × *APP/PS1 Interaction*, F(1, 39) = 4.19, p = 0.047). Indeed, although all females exhibited an increase in freezing in response to the presentation of the tone (*Tone*, F(1, 39) = 145.2, p < 0.0001), female *Slc11a2*^KD^ *APP/PS1* mice were significantly less responsive compared to all other groups (Fig. 5H; *Interaction of Knockdown* × *APP/PS1*, F(1, 39) = 5.39, p = 0.026).

**Figure 6:**
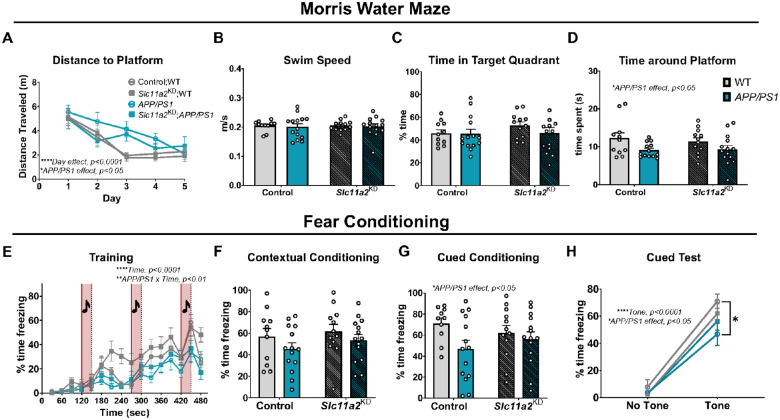
Microglial *Slc11a2* knockdown has no effect on memory performance in male mice. **A-D)** Morris water maze (MWM). **A** Total distance traveled (m) before reaching hidden platform over course of five training days in control WT, control *APP/PS1*, *Slc11a2*^KD^ WT, and *Slc11a2*^KD^ *APP/PS1* male mice. Four trials of 60 s each were conducted each day and averaged per animal. Three-way ANOVA, ****p<0.0001 effect of day, *p<0.05 effect of *APP/PS1*. **B** Average speed (m/s) measured during probe trial. Two-way ANOVA, *p<0.05 effect of *APP/PS1* genotype. ^##^p<0.01 *Slc11a2*^KD^ vs. Control. **C** Total percent time spent in the target quadrant in probe trial for memory. **D** Total time (s) spent around where the platform previously was (exact platform location + 1.5 cm radius) during probe trial for memory. **E-H)** Fear conditioning assay. E Percent component time freezing during the 8 min training protocol. Every 2 min, a 30 s tone was played, followed by a mild foot-shock. Increased freezing behavior over the course of the assay is shown. ****p<0.0001 effect of time, **p<0.0001 effect of *APP/PS1* × time. **F** Percent time freezing during 4 min contextual fear conditioning test. **G** Total percent time spent freezing during the 4 min of cued fear conditioning testing. **H** Percent component time spent freezing during 2 min of no-tone versus 2 min of tone presentation in cued fear conditioning test. *p<0.05 effect of *APP/PS1* genotype, ****p<0.0001 effect of tone. Data represent the mean ± S.E.M. of 11–14 mice per group. Statistical outliers were removed using the Grubb’s test.

Male *APP/PS1* animals displayed a significant deficit in nest building capacity compared to littermate WT control mice, with no additional effect due to *Slc11a2*^KD^ (Control WT mean, 3.17 g ± 0.52; Control *APP/PS1* mean, 2.03 g ± 0.41; *Slc11a2*^KD^ WT mean, 3.82 g ± 0.34; *Slc11a2*^KD^ *APP/PS1* mean, 2.35 g ± 0.44; *APP/PS1 effect*, F(1, 47) = 9.15, p = 0.004). In the MWM, all males regardless of experimental group learned the location of the platform by the end of five training days, albeit *APP/PS1* males exhibited longer path lengths over the course of the training compared to WT controls (Fig. 6A; Males: *Day effect*, F(2.891, 135.9) = 20.45, p < 0.0001; *APP/PS1* effect, F(1, 47) = 5.99, p = 0.018). This behavioral phenotype was observed in the absence of differences in swim speeds between groups (Fig. 6B, p > 0.05), demonstrating a disease model-associated learning deficit in the males. In the MWM probe trial, there were no significant differences between groups in time spent in the target quadrant of the previous platform location (Fig. 6C, p > 0.05); however, male *APP/PS1* mice overall spent significantly less time around the remembered platform location (platform location, including 1.5 cm surrounding radius) compared to WT controls (Fig. 6D; Males: *APP/PS1 effect*, F(1, 46) = 6.55, p = 0.01). There were no differences in male *Slc11a2*^KD^ animals compared to *Slc11a2*-intact control animals in MWM. In the fear conditioning task, male *APP/PS1* animals exhibited decreased freezing during the training session (Fig. 6E, *Interaction of Time* × *APP/PS1*, F(15, 705) = 2.25, p = 0.004). There were no significant differences between any groups of the males in the contextual fear conditioning assay (Fig. 6F, p > 0.05), although male *APP/PS1* mice overall performed worse on the cued fear conditioning task for memory compared to WT controls (Fig. 6G–H, *APP/PS1 effect*, F(1, 46) = 4.15, p = 0.047). *Slc11a2* knockdown had no effect on performance in these assays in the males. Overall, these data suggest that microglial *Slc11a2* knockdown is associated with significant worsening of cognitive dysfunction in several tasks in a sex-specific manner, particularly in female *APP/PS1* animals.

### Hippocampal microglia from female *Slc11a2*^KD^ *APP/PS1* animals exhibit significant alterations in DAM-like inflammatory and oxidative gene markers.

**Figure 7:**
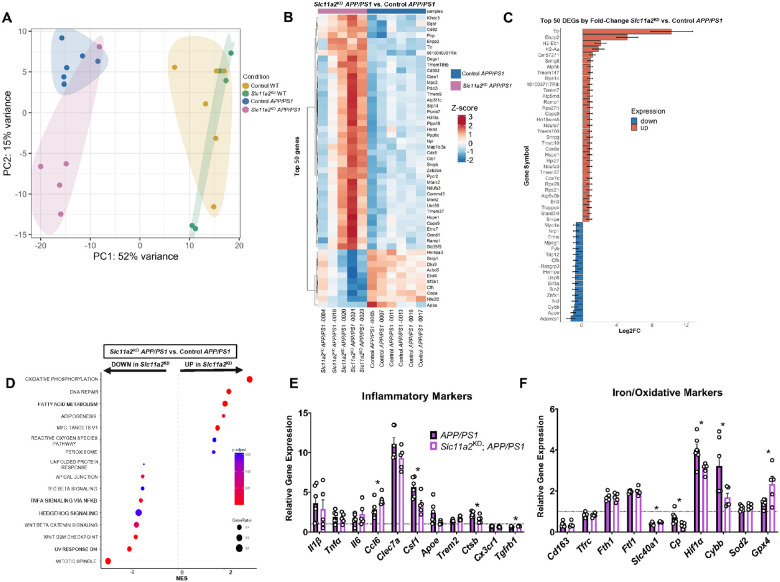
*Slc11a2* knockdown shifts transcriptional profile and alters DAM-related gene markers in hippocampal microglia from female *APP/PS1* mice. **A)** Principal component analysis (PCA) of bulk RNA-seq gene expression in sorted CD11b^+^ microglia from control WT, control *APP/PS1*, *Slc11a2*^KD^ WT, and *Slc11a2*^KD^ *APP/PS1* mice. Primary differences in overall gene expression are a result of *APP/PS1* genotype. **B)** Heat map of top 50 DEGs (by adjusted p-value in RNA-seq dataset) between *Slc11a2*^KD^ *APP/PS1* versus control *APP/PS1* microglia. Blue = downregulated in *Slc11a2*^KD^ cells, lighter blue and/or red = upregulated in *Slc11a2*^KD^ cells. **C)** Top 50 DEGS by fold-change in RNA-seq analysis between *Slc11a2*^KD^ *APP/PS1* and control *APP/PS1* microglia. Red = upregulated in *Slc11a2*^KD^, blue = downregulated in *Slc11a2*^KD^ cells. **D)** GSEA analysis of hallmark gene pathways significantly altered between *Slc11a2*^KD^ *APP/PS1* and control *APP/PS1* microglia. **E-F)** Relative gene expression of targeted **E** inflammatory markers and **F** iron and oxidative stress markers from *Slc11a2*^KD^ *APP/PS1* versus control *APP/PS1* microglia in the RNA-seq dataset. Gene expression is relative to control WT average (black dotted line set to 1). *p<0.05, student’s t-test comparing *Slc11a2*^KD^ *APP/PS1* vs. control *APP/PS1*. Data represent the mean ± S.E.M. of 5–6 mice per group.

Significant alterations in gene expression from isolated microglia have been shown in AD models and human patients (35, 36). Thus, to examine transcriptomic changes in microglia in our studies, we magnetically isolated CD11b + microglia from the bilateral hippocampus from female mice for bulk RNA-sequencing. Primarily, we sought to determine changes in hippocampal microglia that may underlie the behavioral and memory-associated deficits observed primarily in the female *Slc11a2*^KD^ *APP/PS1* animals. In the *Slc11a2* knockdown microglia, we first confirmed abrogation of expression in the *Slc11a2* gene between exons 6–8 (**Additional File 4: Fig. S2A**), similar to what has been shown by others in this mouse model used to knockdown *Slc11a2* (76, 77). Principal component analysis revealed a primary effect of *APP/PS1* genotype on overall gene expression in isolated cells (Fig. 7A). As expected, hippocampal microglia isolated from *APP/PS1* control animals exhibited a significant and robust pattern of differential gene expression compared to microglia isolated from WT controls. We found 1,301 differentially expressed genes (DEG) that were elevated in microglia from *APP/PS1* control animals and 1,236 genes that were significantly downregulated in *APP/PS1* controls compared to WT controls (adjusted p-value < 0.05). In examining the top 50 DEG (by fold-change and adj. p-value) in hippocampal microglia isolated from *APP/PS1* compared to WT control females, we observed changes in similar gene markers previously reported in AD-associated microglia. Specifically, there were robust increases in microglial phagocytic marker *Cd68* (78), hypoxia-related gene *Hif1α* (79), aging-associated marker *Clec7a* (80), lipid-droplet-associated marker *Plin2* (81), as well as Type I IFN-signaling gene, *Mamdc2* (82) (**Additional File 5: Fig. S3A**). DEGs that were downregulated in *APP/PS1* hippocampal microglia compared to WT controls included homeostatic microglial marker *Tmem119*, as well as iron export gene, *Slc40a1* (ferroportin) (**Additional File 5: Fig. S3B**). Gene-set enrichment analysis (GSEA) revealed significant upregulations in genes involved in cholesterol homeostasis, cellular metabolism, and inflammatory activation in *APP/PS1* microglia (**Additional File 5: Fig. S3C**), similar to what others have shown previously in AD models (83).

To determine the effect of *Slc11a2* knockdown on hippocampal microglia, we first compared microglial gene expression between *Slc11a2*^KD^ and Control WT females. As a result of knockdown alone, we only found 7 DEGs (**Additional File 6: Fig. S4A**). Top genes altered included *Ccr6* and *Cd5* (**Additional File 6: Fig. S4B-C**). We then aimed to determine how *Slc11a2* knockdown affects microglial gene expression in the *APP/PS1* female animals. There were 150 genes significantly upregulated and 484 downregulated genes in microglia isolated from *Slc11a2*^KD^ *APP/PS1* animals compared to microglia from control *APP/PS1* mice. Of these *DEGs, Enpp2* and *Ttr* were robustly upregulated in knockdown cells compared to controls (Fig. 7B). Of the top 50 identified DEGs between *Slc11a2*^KD^ *APP/PS1* and control *APP/PS1* females, *Apoe* (encoding apolipoprotein E), *Cybb* (gene for NOX2), and homeostatic marker *Bin2* were also significantly downregulated in the knockdown cells compared to the control *APP/PS1* cells (Fig. 7B–C). GSEA in the *Slc11a2*^KD^ and control *APP/PS1* microglia revealed significant increases in genes associated with cellular metabolism – in particular, oxidative phosphorylation and fatty acid metabolism – and reactive oxygen species (ROS) pathways (Fig. 7D). *Slc11a2* knockdown cells also exhibited significant decreases in genes associated with TNF and NFκB inflammatory signaling and Wnt signaling. When comparing relative expression of specific genes in the sequencing dataset, we observed significant alterations in several genes involved in inflammatory and iron-related pathways in *Slc11a2*^KD^ versus control cells from *APP/PS1* females. Specifically, we observed a significant decrease in DAM markers *Ctsb* and *Csf1* (84) in the knockdown cells, as well as a significant increase in *Tgfbr1* (p < 0.05) and increase in *Trem2* compared to control cells (although not statistically significant, p = 0.068) (Fig. 7E). In examining genes related to iron handling and redox status, we observed a significant increase in iron exporter gene *Slc40a1* and antioxidant gene *Gpx4* in *Slc11a2*^KD^ *APP/PS1* cells compared to control *APP/PS1* microglia (Fig. 7F). Additionally, *Slc11a2* knockdown cells exhibited decreases in pro-oxidant genes, such as *Hif1α* and *Cybb*, and a robust decrease in the iron-related gene encoding ceruloplasmin (*Cp*) (Fig. 7F). Although *Slc11a2*^KD^ cells isolated from *APP/PS1* mice exhibited significant differences in the expression of several DAM markers compared to control *APP/PS1* microglia, *Slc11a2*^KD^ *APP/PS1* microglia displayed a transcriptional profile still distinct from control, non-*APP/PS1* WT cells (black dotted line, Fig. 7E, F). In comparison to control WT cells, *Slc11a2*^KD^ *APP/PS1* microglia upregulated DAM and aging-related markers *Csf1*, *Hif1α*, *Cybb*, and *Ctsb* – albeit, to a lesser degree than control *APP/PS1* microglia.

Initial assessment of overall gene expression via PCA and DEGs in these samples revealed significant variance in gene expression in one sample in the *Slc11a2*^KD^ *APP/PS1* group compared to the rest of the *Slc11a2*^KD^ *APP/PS1* biological replicates (sample labeled as −0004 in PCA plot shown in **Additional File 4: Fig. S2B** and in heat map Fig. 7B). Although this sample was not considered to be a statistical outlier, further RNA-seq analyses conducted following the removal of this sample are shown in **Additional File 7: Fig. S5.** In this analysis, there were 2,210 genes significantly upregulated and 2,230 significantly downregulated in the *Slc11a2*^KD^ versus control *APP/PS1* females (**Additional File 7: Fig. S5B**). The top DEGs revealed upregulations in genes including phagocytic-associated *Igkc*, along with *Ttr* and *Enpp2* (**Additional File 7: Fig. S5C and D**). *Slc11a2*^KD^ cells also exhibited significant downregulations in heat-shock-related genes *Hspa1a* and *Hspa1b*, as well as in inflammatory-related genes *Lag3*, *Inpp5d*, *Erap1*, and *H2-Aa*, and in LDAM (lipid-droplet-accumulating microglia) marker *Ly9* (**Additional File 7: Fig. S5C and D**). Overall, these data suggest that microglial *Slc11a2* knockdown in females decreases the DAM-like and aging-associated transcriptional signatures in the *APP/PS1* model.

## Discussion

Iron-loaded microglia are a hallmark of several neurodegenerative diseases, including AD (85–87). Reactive microglia surrounding Aβ plaques exhibit a significant upregulation in ferritin-L (Ftl1) across AD mouse models and human patients and is thus a defining feature of DAMs across multiple disease models (31, 35, 36, 88). Furthermore, recent *in vitro* work showed that iron loading specifically in microglia underlies subsequent neurotoxicity and cell death in a tri-culture system, positioning microglial iron load as a central mediator of neurodegeneration (29). At the *in vitro* level in microglia, inflammatory signals and iron import mechanisms are intimately connected [(20, 24), our data also in IMG cells]. Increased iron levels have been shown to enhance pro-inflammatory cytokine secretion (45), toxic ROS production (40), and cellular senescence and dysfunction (75, 89). Reciprocally, AD-associated pro-inflammatory signals such as Aβ and bacterial lipopolysaccharide (LPS) upregulate microglial iron importer DMT1 (gene, *Slc11a2*).

In our studies in primary microglia from aged and young mice treated *in vitro* with pro-inflammatory oligomeric Aβ_1–42_, we observed that the Aβ-induced increase in *Slc11a2* was exacerbated in microglia from aged compared to young mice. This age-associated increase in *Slc11a2* expression was accompanied by a significant upregulation in iron storage genes *Ftl1* and *Fth1* and augmented Aβ-induced inflammatory markers. An exacerbated Aβ-provoked inflammatory response in aged glia has been similarly observed by others and suggests a primed cellular state (90, 91). Our findings demonstrate an association between augmented Aβ-induced inflammation and iron loading markers in aged cells and suggest a synergy between age and Aβ leading to increased microglial *Slc11a2* expression. Considering the purported role of iron in promoting cellular senescence and inflammation, it may be that DMT1/*Slc11a2* plays a role in mediating the concomitant cellular iron and inflammatory load observed in neurodegenerative disease. Indeed, a role for DMT1 in Parkinson’s disease is well-appreciated (92, 93). However, no studies to our knowledge have directly examined whether altering microglial DMT1/*Slc11a2 in vivo* affects the development of chronic inflammation and disease-associated hallmarks in AD.

To investigate the effects of cell-specific alteration of *Slc11a2* in AD, we generated a novel model of tamoxifen-inducible, microglial-specific knockdown of *Slc11a2* in the *APP/PS1* mouse model of AD. In female *Slc11a2*^KD^ *APP/PS1* mice, we observed a significant worsening of behavioral phenotypes and cognitive performance at 12–15 months of age following *Slc11a2* knockdown at 5–6 months of age. Specifically, female *Slc11a2*^KD^ *APP/PS1* animals were significantly more hyperactive than all other female groups in multiple assays conducted. Previous studies have demonstrated significant hyperactivity in mouse models of AD (94–97), and AD human patients often exhibit disruptions in psychiatric behaviors such as hyperactivity, impulsivity, and disinhibition (98). Thus, microglial *Slc11a2* knockdown exacerbates disruptions in these neuropsychiatric-like symptoms in our model, particularly in female *APP/PS1* mice.

To assay for changes in memory function, we utilized the MWM test – a gold standard for testing hippocampal-dependent spatial memory acquisition and retention in rodents (58, 99). *Slc11a2* knockdown resulted in a significant worsening of memory function in *APP/PS1* females in the MWM memory probe trial. To further probe this memory phenotype, we used a fear conditioning assay consisting of both a contextual conditioning and cued conditioning task. We observed a significant deficit in learned cued fear memory in female *Slc11a2*^KD^ *APP/PS1* mice compared to control WT and *APP/PS1* mice. The cued fear conditioning task utilizing the re-presentation of a cue (tone previously paired with a shock) requires the use of separate, parallel neural processing systems from the contextual fear memory task, involving inputs from the amygdala, insular cortex, regions in the parietal and temporal lobes, sensory cortices, and thalamus (100, 101). These complex networks likely converge with hippocampal circuits to acquire and express fear memory associated with a conditioned stimulus (102). Interestingly, dysfunction and neurodegeneration in the amygdala (103–105) and insular cortex (106, 107) have been implicated in AD models and patients as an early indicator of disease, and may also underlie many of the neuropsychiatric symptoms observed in AD patients, such as hyperactivity and agitation (108). The deficits we observed in cued fear memory in the female *Slc11a2*^KD^ *APP/PS1* mice, paired with their significant hyperactivity, suggest that *Slc11a2* knockdown may worsen AD-associated behavior mediated by both hippocampal and non-hippocampal-dependent circuits in female mice. These data thus reflect a sex-specific, disease-modifying cognitive effect of *Slc11a2* knockdown in female, but not male, *APP/PS1* mice.

The sex-specific effects of microglial *Slc11a2* knockdown found in our work are of particular interest in relation to AD development. In humans, females are significantly more likely to develop AD than males (109, 110), and female mice display enhanced pathological hallmarks compared to males in AD models (111–114). In the studies reported here, we observed effects of microglial *Slc11a2* knockdown in female, but not male, *APP/PS1* mice, suggesting a potential pathway involved in worsening disease parameters in female mice. Sex differences in brain iron-handling and changes in iron-associated markers related to disease development are not well understood. In humans, brain ferritin levels are generally higher in older men than women in several regions (115), which is thought to contribute to the risk for males developing neurodegenerative disease at earlier ages than females (116). In females, but not males, prior iron-deficiency anemia is associated with the development of dementia in females (117). On the other hand, there is a significant rise in serum ferritin levels associated with menopause in aging females (118), and this rise in iron status during female mid-life has been directly correlated with declining cognitive performance (119). In mice, males have higher brain iron levels than females (120), and recent work showed that adult male and female mice differentially alter brain iron stores in iron-deficient conditions (121). Additionally, research has illuminated significant sex differences in microglial morphology, inflammatory markers, and activity in age and disease, which may also contribute to sex differences in AD development (122, 123). Although the exact associations between brain iron status, sex, and microglial function are still being elucidated, our work suggests that a particular microglial inflammatory-iron-related pathway may be relevant to sex-dependent differences in inflammation and disease progression.

Although we were technically limited to gene expression analyses in these studies, future work aimed at characterizing iron load in the *Slc11a2*^KD^ cells would be needed to expand upon these findings. While we cannot make definitive conclusions related to iron levels and/or protein-level changes in DMT1 and inflammatory makers per se, others have demonstrated an important role for transcription-level changes in inflammatory and iron-related genes (124, 125). Furthermore, many studies have characterized the microglial transcriptional landscape during AD (80, 126, 127). We conducted RNA-seq on isolated hippocampal microglia from the female mice in these studies to assess transcriptional changes that may underlie the cognitive differences observed. We found robust increases in *Enpp2* and *Ttr* in *Slc11a2*^KD^ *APP/PS1* microglia compared to controls. Although there is a possibility these genes are associated with choroid plexus contamination in the hippocampal samples (128, 129), previous work in models of neuroinflammation and AD has identified downregulations in the expression of *Enpp2*, or ectonucleotide pyrophosphatase 2, and *Ttr*, the gene encoding for transthyretin, in specific DAM subsets (130–132). These two genes play roles in protein folding, Aβ binding, and lipid signaling, and have been suggested to play significant deleterious roles in microglia during aging and disease, when they are increased (133, 134).

*Slc11a2* ^KD^ cells from *APP/PS1* females also exhibited decreases in DAM-related and age-associated markers, such as *Apoe, Csf1, Cybb*, and *Ctsb*. Upregulations in these genes in AD-associated microglia are thought to be representative of a ‘primed’ microglial expression state during disease pathogenesis (132). While much work remains to elucidate the specific functions of heterogeneous microglial populations during AD, this DAM-like phenotype is posited to be a protective cell state initiated in response to growing AD pathology (35, 135, 136). Thus, decreases in these markers in the *Slc11a2*^KD^ cells may reflect the loss of a protective transcriptional state. *Slc11a2*^KD^ cells from *APP/PS1* females also displayed a significant decrease in *Bin2*, a marker related to cell migration and phagocytosis, and decreases in oxidative stress regulators *Hif1α* and *Nfe2l2*. A decrease in *Bin2* expression has been shown to mark a disease-promoting transition in microglia during AD progression (137, 138), and increases in *Hif1α* and *Nfe2l2* in AD are thought to be a response to prevent excessive oxidative damage and microgliosis in disease (36, 139, 140). Our data thus suggest that *Slc11a2* knockdown in microglia during AD progression leads to a lessening of the appearance of ‘protective’ DAM-related markers associated with limiting cellular damage (i.e., *Apoe, Hif1α, Csf1, Nfe2l2*), and instead an exacerbation of deleterious transcriptional changes observed in aged and AD-associated microglia (i.e., *Bin2, Enpp2*). Interestingly, although microglial *Slc11a2* knockdown improved inflammatory markers and sickness in our acute LPS model (42), it may be that a shift in these inflammatory pathways in microglia in the *APP/PS1* model is an important defensive measure in the context of chronic disease and long-term cognitive protection.

With the exception of differences in iron export gene, *Slc40a1*, and ceruloplasmin gene, *Cp*, there were few significant changes in iron-associated genes in *Slc11a2*^KD^ cells in our RNA-seq data. This could suggest that the effect of *Slc11a2* knockdown is primarily on inflammatory markers and not on iron markers per se, or it could reflect a time-dependent transcriptional change in those markers during AD progression that was not captured at the time point tested. Our *in vitro* work utilizing ebselen to inhibit DMT1 demonstrated that at the cellular level, ebselen robustly decreases Aβ-induced inflammatory markers and alters iron load. Ebselen functions as both a potent DMT1 inhibitor (63) and is also a peroxidase mimetic, and thus holds potential as a therapeutic to simultaneously limit cellular iron uptake, ROS production, and inflammatory signaling (141–143). Indeed, ebselen can improve phenotypes in AD models, and this may be due in part to its effects exerted on DMT1 and iron-handling (144, 145). While our *in vivo* work cannot definitively demonstrate that the effects of DMT1 knockdown are due to differences in iron load per se, it is intriguing to note that the directional changes in inflammatory and oxidative markers in microglia from the *Slc11a2*^KD^ females mimic the anti-inflammatory and antioxidant effects of ebselen. While ebselen targets several pathways in the cell, it may be that some of the anti-inflammatory and antioxidant effects of ebselen are due in part to DMT1 inhibition (146, 147). Future work is needed to measure levels of iron in knockdown cells to conclusively determine the cellular effects of knockdown. However, even if total cellular iron levels do not change due to *Slc11a2* knockdown, it could be that alterations in DMT1 affect the localization or distribution of iron in the cell that then alters inflammatory signaling.

Lastly, it is intriguing to consider a different time point for manipulation of microglial *Slc11a2* during the disease process or for microglial collection for analysis. It may be that microglial increases in iron-related markers are initially a neuroprotective measure, whereas a late transition to an iron-import transcriptional phenotype results in exceeding the cell’s capacity for non-toxic iron-handling and leads to neurodegenerative consequences (29, 148).

## Conclusions

In conclusion, this work highlights a sex-specific effect of microglial knockdown of iron import gene *Slc11a2* on behavior and cognitive function in the *APP/PS1* mouse model of AD. Female *Slc11a2*^KD^ *APP/PS1* mice are significantly more hyperactive and display worsened memory phenotypes compared to control animals. Associated with these behavioral changes, microglia from *Slc11a2*^KD^ *APP/PS1* females display a transcriptional shift demonstrating decreased DAM markers purported to be protective. These data suggest that knockdown of iron import gene, *Slc11a2*, leads to a progressive worsening of disease parameters in female AD mice and illuminate a microglial inflammatory-iron-associated pathway that holds relevance to our understanding of the complex roles of iron and microglia in neurodegenerative disease.

## Data Availability

The datasets supporting the conclusions of this article are available in the NCBI Gene Expression Omnibus (GEO) repository with accession ID GSE269314 (https://www.ncbi.nlm.nih.gov/geo/query/acc.cgi?acc=GSE269314). Other datasets used and/or analyzed are available from the corresponding authors on reasonable request. Additionally, sperm from *Slc11a2*^flfl^;*Cx3cr1*^Cre-ERT2^;*APP/PS1*^+^ triple-transgenic male mice (129S;C57BL/6J) were cryopreserved at the Vanderbilt Genome Editing Resource to preserve the genetic line used in these studies, and are available for sharing upon request.

## Abbreviations

Aβ: amyloid-beta
AD: Alzheimer’s disease
*Apoe*: apolipoprotein E
*APP/PS1*: APPswe,PSEN1dE9
CNS: central nervous system
DAM: disease-associated microglia
DEG: differentially expressed genes
DMSO: dimethyl sulfoxide
DMT1: divalent metal transporter 1
EZM: elevated zero maze
FAC: ferric ammonium citrate
Fth: ferritin heavy chain
Ftl: ferritin light chain
GSEA: gene set enrichment analysis
*Hif1α*: hypoxia-inducible factor 1 α
ICP-MS: inductively-coupled plasma mass spectrometry
IL: interleukin
IMG: immortalized microglial cell line
KD: knockdown
MWM: Morris water maze
PCA: principal component analysis
ROS: reactive oxygen species
*Slc11a2*: solute carrier family 11 member 2
*Tnfα*: tumor necrosis factor α
WT: wild-type
